# Activity of ceftolozane/tazobactam against surveillance and ‘problem’ Enterobacteriaceae, *Pseudomonas aeruginosa* and non-fermenters from the British Isles

**DOI:** 10.1093/jac/dkx136

**Published:** 2017-05-16

**Authors:** David M. Livermore, Shazad Mushtaq, Daniele Meunier, Katie L. Hopkins, Robert Hill, Rachael Adkin, Aiysha Chaudhry, Rachel Pike, Peter Staves, Neil Woodford, the BSAC Resistance Surveillance Standing Committee

**Affiliations:** 1 Antimicrobial Resistance and Healthcare Associated Infections (AMRHAI) Reference Unit, National Infection Service, Public Health England, London, UK; 2 Norwich Medical School, University of East Anglia, Norwich, Norfolk, UK

## Abstract

**Background:** We assessed the activity of ceftolozane/tazobactam against consecutive isolates collected in the BSAC Bacteraemia Surveillance from 2011 to 2015 and against ‘problem’ isolates sent to the UK national reference laboratory from July 2015, when routine testing began.

**Methods:** Susceptibility testing was by BSAC agar dilution with resistance mechanisms identified by PCR and interpretive reading.

**Results:** Data were reviewed for 6080 BSAC surveillance isolates and 5473 referred organisms. Ceftolozane/tazobactam had good activity against unselected ESBL producers in the BSAC series, but activity was reduced against ertapenem-resistant ESBL producers, which were numerous among reference submissions. AmpC-derepressed *Enterobacter* spp. were widely resistant, but *Escherichia coli* with raised chromosomal AmpC frequently remained susceptible, as did *Klebsiella pneumoniae* with acquired DHA-1-type AmpC. Carbapenemase-producing Enterobacteriaceae were mostly resistant, except for ceftazidime-susceptible isolates with OXA-48-like enzymes. Ceftolozane/tazobactam was active against 99.8% of the BSAC *Pseudomonas aeruginosa* isolates; against referred *P. aeruginosa* it was active against 99.7% with moderately raised efflux, 94.7% with strongly raised efflux and 96.6% with derepressed AmpC. Resistance in *P. aeruginosa* was largely confined to isolates with metallo-β-lactamases (MBLs) or ESBLs. MICs for referred *Burkholderia* spp. and *Stenotrophomonas maltophilia* were 2–4-fold lower than those of ceftazidime.

**Conclusions:** Ceftolozane/tazobactam is active against ESBL-producing Enterobacteriaceae; gains against other problem Enterobacteriaceae groups were limited. Against *P. aeruginosa* it overcame the two most prevalent mechanisms (up-regulated efflux and derepressed AmpC) and was active against 51.9% of isolates non-susceptible to all other β-lactams, rising to 80.9% if ESBL and MBL producers were excluded.

## Introduction

Ceftolozane/tazobactam is a cephalosporin/β-lactamase inhibitor combination, recently licensed for complicated intra-abdominal and urinary tract infections.^1^ EUCAST breakpoints, with a fixed 4 mg/L concentration of tazobactam, are susceptible ≤1 mg/L/resistant >1 mg/L for Enterobacteriaceae and ≤4/>4 mg/L for *Pseudomonas aeruginosa*. Tazobactam protects ceftolozane against ESBLs, with the combination’s efficacy against producers confirmed in the Phase III trials.^1^ Ceftolozane is also notably active against *P. aeruginosa*, with MICs lower than ceftazidime—hitherto the most active anti-pseudomonal β-lactam.^2^ This activity is retained for many strains with derepressed AmpC or up-regulated efflux,^2^ which are the major routes to resistance to established penicillins and cephalosporins in the species.^3^

Here, we review the activity of ceftolozane/tazobactam against two large, contrasting series of Gram-negative isolates. First, we considered consecutive bloodstream isolates collected by the BSAC Bacteraemia Surveillance during 2011–15. Secondly, we reviewed isolates referred to PHE’s Antimicrobial Resistance and Healthcare Associated Infections (AMRHAI) Reference Unit in the first year that ceftolozane/tazobactam was tested; thereby providing a large snapshot of UK ‘problem’ organisms.

## Materials and methods

### BSAC surveillance

The BSAC Bacteraemia Surveillance has been described previously.^4^ Results were reviewed from 2011, when testing of ceftolozane/tazobactam began, to 2015; 38–40 UK and Irish laboratories contributed annually, submitting up to seven consecutive bloodstream isolates each of *Klebsiella*, *Enterobacter* and *Pseudomonas* spp. and up to 14 *Escherichia coli*. Identifications were confirmed at AMRHAI by MALDI-TOF or API20E or API20NE strips (bioMérieux, Marcy-l’Étoile, France). Among *Pseudomonas* spp., only confirmed *P. aeruginosa* were considered; these comprised 95.1% of all *Pseudomonas* spp. collected. Susceptibility testing was by BSAC agar dilution.^5^ Enterobacteriaceae with cefotaxime or ceftazidime MICs ≥1 mg/L were further characterized by: (i) comparing cefepime, cefotaxime and ceftazidime MICs with and without 4 mg/L clavulanate to identify ESBL producers (with ≥8-fold potentiation of *≥*1 cephalosporin); (ii) comparing cefotaxime MICs with and without 100 mg/L cloxacillin to identify AmpC producers (with ≥4-fold potentiation, no cephalosporin/clavulanate potentiation, and ≥8-fold higher MICs of cefotaxime and ceftazidime than cefepime); and (iii) by PCR for *bla*_CTX-M_ and plasmid AmpC enzymes. Enterobacteriaceae with reduced carbapenem susceptibility were screened for carbapenemase genes by PCR, as were *P. aeruginosa* that were both (i) broadly resistant to β-lactams and (ii) positive for imipenem/EDTA synergy.

### Reference submissions

MICs, determined by BSAC agar dilution,^5^ were reviewed for all non-fastidious Gram-negative bacteria referred to PHE’s AMRHAI Reference Unit over 1 year from July 2015, when routine testing of ceftolozane/tazobactam began. Around 90% of submission are from diagnostic laboratories in England, 9% from elsewhere in the UK and 1% from overseas, principally Ireland. We excluded isolates tested for internal and external quality assurance and repeat/multiple tests on the same isolate from the same submission. Testing employed a wide panel of antibiotics. To predict β-lactamase types, MICs of cefotaxime/clavulanate 2 mg/L, cefotaxime/cloxacillin 100 mg/L, ceftazidime/clavulanate 2 mg/L, ceftazidime/avibactam 2 mg/L, cefepime/clavulanate 2 mg/L and imipenem/EDTA 320 mg/L were compared with those of the same β-lactams alone.

Genes for KPC, VIM, NDM and OXA-48-like carbapenemases were sought by multiplex PCR. Enterobacteriaceae resistant to meropenem and imipenem but lacking genes for these common carbapenemases were subjected to further multiplex PCRs, seeking (i) *bla*_IMP_, *bla*_VIM_, *bla*_SPM_, *bla*_GIM_, *bla*_SIM_ and *bla*_NDM_ and (ii) *bla*_FRI_, *bla*_GES_, *bla*_IMI_ and *bla*_SME_. Metallo-carbapenemase genes were sought in *P. aeruginosa* isolates showing ≥8-fold or greater imipenem/EDTA synergy together with broad resistance to penicillins and cephalosporins. *bla*_VEB_ and *bla*_PER_ genes were sought by PCR in most *P. aeruginosa* isolates with ceftazidime MICs >256 mg/L and ceftazidime/clavulanate MICs ≤32 mg/L. Referred *Acinetobacter* spp. isolates were screened for *bla*_OXA-51_-like to identify *A. baumannii* where this gene is universal and chromosomal.

### Categorization of isolates

Detection of a β-lactamase gene was considered ‘definitive’ identification of a mechanism. Carbapenemase-negative isolates were categorized by in-house mathematical algorithms employing the principles of interpretive reading of phenotypes.^6^ Two levels of match were allowed: ‘hard’, where the phenotype was a perfect match; and ‘soft’, where the phenotype was less perfectly matched, but the mechanism remained the most likely. For example, to meet hard-match criteria for highly raised (putatively MexAB-OprM-mediated) efflux, a *P. aeruginosa* isolate required a carbenicillin MIC >512 mg/L with carbencillin:cefotaxime:piperacillin:ceftazidime MIC ratios in the ranges 1:0.25–1:0.03–0.12:0.015–0.06 (note these are ratios, not actual MICs, and are predicated on the fact that up-regulated MexAB-OprM raises MICs of these agents in rough proportion);^4^ to meet soft-match criteria the strain needed a carbenicillin MIC >512 mg/L and carbenicillin:cefotaxime:piperacillin:ceftazidime MIC ratios in the ranges 1:0.12–2:0.015–0.25:0.008–0.12. Some isolates did not match any of the phenotypes considered and were left as ‘unassigned’, then categorized according to their level of resistance to reference agents, principally ceftazidime.

## Results

### BSAC Bacteraemia Surveillance isolates

The BSAC collection provided a random and geographically diverse sample of bloodstream isolates. Because no temporal trend was seen for ceftolozane/tazobactam (not shown), data for 2011 to 2015 were pooled (Table 1). Using EUCAST’s 1 + 4 mg/L Enterobacteriaceae breakpoint, susceptibility rates to ceftolozane/tazobactam were 91.5% for *Enterobacter* spp., 97.6% for *Klebsiella* spp. and 99.7% for *E. coli*, exceeding those for all other β-lactams tested except carbapenems. In the case of *P. aeruginosa*, 99.8% of isolates (1097/1099) were susceptible at EUCAST's 4 + 4 mg/L breakpoint, versus 97.9% for gentamicin, 97.6% for ceftazidime, 95.5% for piperacillin/tazobactam, 91.7% for meropenem (not tested in 2012 and 2013), 92.5% for imipenem (not tested in 2014) and 90.4% for ciprofloxacin.

**Table 1:** MIC distributions of ceftolozane/tazobactam for *E. coli, Klebsiella* spp., *Enterobacter* spp. and *P. aeruginosa* in the BSAC Bacteraemia Surveillance Programme, 2011–15 inclusive

	No. of isolates with indicated ceftolozane/tazobactam MIC (mg/L)																
Organism	≤0.03	0.06	0.12	0.25	0.5	1	2	4	8	16	32	64	128	256	>256	Total	%S^a^
All																	
* E. coli*	2	67	1710	775	94	20	5		3							2676	99.7
* Enterobacter* spp.	2	18	160	568	114	61	27	26	22	8	2	1				1009	91.5
* Klebsiella* spp.		12	338	662	192	61	14	2	2	1	1	4	2	4	1	1296	97.6
* P. aeruginosa*			7	242	756	82	6	4	1		1					1099	99.8
ESBL (all)																	
* E. coli*		1	42	151	66	15	4		2							281	97.9
* Enterobacter* spp.			3	17	14	6	4		1	1	1					47	85.1
* Klebsiella* spp.			4	11	57	28	9		1	1	1	3	1	3		119	84.0
CTX-M group 1 ESBLs^b^																	
* E. coli*			29	133	54	12	3		2							233	97.9
* Enterobacter* spp.				3	7	4	2									16	87.5
* Klebsiella* spp.			3	7	47	14	2		1		1	3	1	3		82	86.6
AmpC																	
* E. coli*			6	16	5	1	1									29	96.6
* Enterobacter* spp.			3	14	20	35	20	24	20	6	1	1				144	50.0
ESBL + AmpC																	
* Enterobacter*				7	4	4	2	1	1	1						20	75
* Klebsiella* spp.				1												1	1/1
Others																	
K1 *K. oxytoca*			1	8	9	9	2									29	93.7
KPC *Klebsiella*							1					1				2	0/2
VIM *Klebsiella*													1	1	1	3	0/3
OXA-48 Enterobacteriaceae						3	1		2							6	3/6

a S, susceptible. Based on a breakpoint of 1 + 4 mg/L for Enterobacteriaceae and 4 + 4 mg/L for *P. aeruginosa*; percentage susceptibility is cited if > 10 isolates, otherwise the proportion of isolates found susceptible is shown.

b Excluding isolates also found to have carbapenemase and/or AmpC activity.

Almost all ESBL- and AmpC-producing *E. coli* (97.9% and 96.6%, respectively) were susceptible to ceftolozane/tazobactam 1 + 4 mg/L, as were 93.7% of *Klebsiella oxytoca* hyperproducing K1 β-lactamase. Susceptibility rates for ESBL-producing *Klebsiella* and *Enterobacter* were lower, at 84%–85%. Around half the ceftolozane/tazobactam resistance among ESBL-producing *Klebsiella* was low level, with MICs of 2 + 4 mg/L; but other isolates were substantially resistant, with MICs up to >256 mg/L. High MICs were not associated with particular ESBL types: 9/13 (69.2%) ESBL-producing isolates with MICs >8 mg/L had CTX-M group 1 enzymes, as did 85/140 (60.7%) with MICs ≤1 mg/L. It is possible that the more resistant isolates had larger amounts of ESBL, a different CTX-M variant, or multiple β-lactamases. Half of the AmpC-hyperproducing *Enterobacter* spp. were resistant; significantly, these ceftolozane/tazobactam-resistant organisms also were much more resistant to cefotaxime and ceftazidime (geometric mean MICs 76.8 and 69.8 mg/L respectively) than were ceftolozane/tazobactam-susceptible AmpC enterobacters (geometric mean cefotaxime and ceftazidime MICs 6.5 and 5.0 mg/L, respectively). It is likely that this variation reflected the amount of AmpC enzyme.

Just 11/4981 (0.2%) BSAC *E. coli*, *Klebsiella* and *Enterobacter* spp. isolates had carbapenemases, 2 with KPC, 3 with VIM and 6 with OXA-48-like enzymes. Three of the six with OXA-48-like enzymes were susceptible to ceftazidime at breakpoint (≤1 mg/L); these also were susceptible to ceftolozane/tazobactam 1 + 4 mg/L. The remaining three OXA-48-like isolates, which were resistant to ceftazidime (MICs 16–128 mg/L), also were resistant to ceftolozane/tazobactam, as were all the isolates with KPC and VIM enzymes.

One of the two ceftolozane/tazobactam-resistant *P. aeruginosa*, with an MIC of 32 + 4 mg/L, had a VIM carbapenemase; mechanisms remain uncertain in the other, with an MIC of 8 + 4 mg/L.

### Isolates referred to PHE

The isolates examined in ceftolozane/tazobactam’s first year of testing comprised 3249 Enterobacteriaceae, 1414 *P. aeruginosa* and 810 other non-fermenters, including 419 *Acinetobacter* spp. They lack a denominator and are referred for numerous reasons. There is a considerable bias towards submitting isolates suspected of carbapenem resistance, but some have suspected colistin, aminoglycoside or tigecycline resistance; a few are referred owing to anomalous susceptibility, e.g. to ampicillin in *P. aeruginosa*. The overall performance data shown in Table 2 should be considered with these biases in mind. Nevertheless two points are striking: first, that ceftolozane/tazobactam was more widely active against these ‘problem’ *P. aeruginosa* than any other β-lactam, whereas, secondly, gains against problem Enterobacteriaceae were modest, with carbapenems remaining more active.

**Table 2:** Overall susceptibilities of Enterobacteriaceae and *P. aeruginosa* referred to AMRHAI

	Enterobacteriaceae (*n* *=* 3249)			*P. aeruginosa* (*n* *=* 1414)		
	BP (mg/L)	no. S	%S	BP (mg/L)	no. S	%S
Colistin	S ≤ 2	2951	90.8	S ≤ 2	1351	95.5
				S ≤ 4	1369	96.8
Amikacin	S ≤ 8	2729	84.0	S ≤ 8	1033	73.1
Tigecycline	S ≤ 1	2449	75.4	—	—	—
Imipenem	S ≤ 2	2365	72.8	S ≤ 4	235	16.6
Gentamicin	S ≤ 2	2062	63.5	S ≤ 4	991	70.1
Meropenem	S ≤ 2	2300	70.8	S ≤ 2	149	10.5
				S ≤ 4	245	17.3
Tobramycin	S ≤ 2	1748	53.8	S ≤ 4	1031	72.9
Ciprofloxacin	S ≤ 0.5	1465	45.1	S ≤ 0.5	535	37.8
Ceftolozane/tazobactam	S ≤ 1 + 4	1048	32.3	S ≤ 4 + 4	1193	84.4
Cefepime	S ≤ 1	947	29.1	S ≤ 8	744	52.6
Temocillin	S ≤ 8	880	27.1	—	—	—
Ertapenem	S ≤ 0.5	731	22.5	—	—	—
Aztreonam	S ≤ 1	678	20.9	—	—	—
Piperacillin/tazobactam	S ≤ 8 + 4	531	16.3	S ≤ 16 + 4	661	46.7
Ceftazidime	S ≤ 1	505	15.5	S ≤ 8	802	56.7
Cefotaxime	S ≤ 1	432	13.3	—	—	—
Amoxicillin/clavulanate	S ≤ 8	118	3.6	—	—	—
Ampicillin	S ≤ 8	18	0.6	—	—	—
Carbenicillin	—	—	—	S ≤ 128	555	39.3

BP, breakpoint; no. S, number susceptible; %S, percentage susceptible.

### Enterobacteriaceae by inferred or proven mechanism

Table 3 shows the MIC distributions of ceftolozane/tazobactam for referred Enterobacteriaceae by species and resistance type. Because there was little difference in their distributions, MICs are pooled for the hard- and soft-matched groups.

**Table 3:** MIC distributions of ceftolozane/tazobactam, by resistance group and species group, for Enterobacteriaceae referred to AMRHAI, July 2015–July 2016

	Matching to mechanism				No. of isolates with indicated MIC (mg/L)									
	molecular	hard^a^	soft^a^	total	≤0.25	0.5	1	2	4	8	16	>16	void	%S at 1 + 4 mg/L BP
ESBL producers														
* Citrobacter* spp.		2		2		1				1				50.0
* E. coli*		337	25	362	100	57	35	38	21	17	32	61	1	53.0
* Enterobacter* spp.		45	4	49	6	4	10	7	4	6	6	6		40.8
* K. oxytoca*			3	3			1	1	1					33.3
* K. pneumoniae*		229	26	255	17	27	23	39	23	24	28	74		26.3
* *rare fermenters		2		2				2						0.0
* Serratia* spp.		1		1								1		0.0
* *all		616	58	674	123	89	69	87	49	48	66	142	1	41.7
AmpC hyperproducers														
* Citrobacter* spp.		39	7	46	3	3	4	6	8	8	7	7		21.7
* E. coli*		101	19	120	35	15	11	11	8	11	7	22		50.8
* Enterobacter* spp.		625	24	649	13	53	53	95	131	115	125	63	1	18.4
* Hafnia alvei*		11	1	12			1		1		2	8		8.3
* K. pneumoniae*		39	10	49	9	6	10		3	6	3	12		51.0
* M. morganii*		7	2	9	3	3	2	1						88.9
* Providencia* spp.		1		1								1		0.0
* *rare fermenters		1		1					1					0.0
* Serratia* spp.		30	4	34		1	16	10	4	1	1	1		50.0
* *all		854	67	921	63	81	97	123	156	141	145	114	1	26.2
Isolates with both ESBL + AmpC activity														
* Citrobacter* spp.		1		1							1	1		0.0
* E. coli*		28	14	42		2	1	1	4	5	29	42		7.1
* Enterobacter* spp.		18	2	20		1	1	4	3	9	2	20		10.0
* K. pneumoniae*		6	2	8	1			1	1		5	8		12.5
* *all		53	18	71										8.5
K1 hyperproducers														
* K. oxytoca*		8		8		3	3	1	1					75.0
KPC producers														
* Citrobacter* spp.	4			4						2	2			0.0
* E. coli*	36			36	1		2	9	14	7	2	1		8.3
* Enterobacter* spp.	28			28		1		3	5	7	8	4		3.6
* K. oxytoca*	4			4			1	1		2				25
* K. pneumoniae*	138			138	1		1	4	44	29	33	26		1.4
* *rare fermenters	2			2							2			0.0
* Serratia* spp.	4			4							3	1		0.0
* *all	216			216	2	1	4	17	63	47	50	32		3.2
GES carbapenemases														
* E. coli*	4			4					1			3		0.0
* Enterobacter* spp.	1			1					1					0.0
* K. oxytoca*	15			15							11	4		0.0
* K. pneumoniae*	3			3						1	1	1		0.0
* *rare fermenters	1			1						1				0.0
* Serratia* spp.	1			1								1		0.0
* *all	25			25					2	2	12	9		0.0
Other class A carbapenemases														
* Enterobacter* spp.	8			8	3	3	1					1		87.5
* Serratia* spp.	4			4		1	1	2						50.0
* *all	12			12	3	4	2	2				1		75.0
MBL producers														
* Citrobacter* spp.	13			13							1	12		0.0
* E. coli*	95			95								95		0.0
* Enterobacter* spp.	29			29								29		0.0
* K. oxytoca*	3			3								3		0.0
* K. pneumoniae*	169			169								169		0.0
* M. morganii*	2			2								2		0.0
* Providencia* spp.	4			4								4		0.0
* Serratia* spp.	1			1								1		0.0
* *all	316			316							1	315		0.0
Isolates with both NDM + OXA-48 carbapenemases														
* E. coli*	4			4								4		0.0
* K. pneumoniae*	28			28								28		0.0
* *all	32			32								32		0.0
OXA-48 producers ceftazidime ≤4 mg/L														
* Citrobacter* spp.	3			3		1	2							100
* E. coli*	74			74	24	33	8	7	1	1				87.8
* Enterobacter* spp.	22			22	1	6	8	7						68. 2
* K. oxytoca*	3			3	1		2							100
* K. pneumoniae*	50			50	2	16	20	9	3					76
* *rare fermenters	1			1		1								100
* Serratia* spp.	2			2			2							100
* *all	155			155	28	57	42	23	4	1				81.9
OXA-48 producers ceftazidime >4 mg/L														
* Citrobacter* spp.	10			10							4	6		0.0
* E. coli*	59			59		4	6	3	9	13	5	19		17.0
* Enterobacter* spp.	23			23			4	3	4	3	5	4		17.4
* K. oxytoca*	4			4						1	2	1		0.0
* K. pneumoniae*	100			100	1	1		3	5	10	8	72		2
* Serratia* spp.	2			2								2		0.0
* *all	198			198	1	5	10	9	18	27	24	104		8.1
Impermeability														
* E. coli*		36		36	6	14	8	4	3	1	6			77.8
* K. oxytoca*		1		1				1						0.0
* K. pneumoniae*		51		51	2	11	28	8	2		2			80.4
* *all		88		88	8	25	36	13	5	1				78.4
WT, cephalosporin susceptible														
* Citrobacter* spp.		2		2	1		1							100
* E. coli*		35		35	34	1								100
* Enterobacter* spp.		47		47	30	14	2	1						97.9
* K. oxytoca*		2		2	1		1							100
* K. pneumoniae*		18		18	17	1								100
* M. morganii*		14		14	12		1	1						92.9
* Providencia* spp.		2		2	1	1								100
* *rare fermenters		6		6	4	2								100
* Serratia* spp.		19		19	4	7	6	1	1					89.5
* *all		145		145	104	26	11	3	1					97.2
Unassigned, ceftazidime MIC ≤4 mg/L														
* Citrobacter* spp.				2	1			1						50
* E. coli*				75	48	13	14							100
* Enterobacter* spp.				12	1	1	8	2						83.3
* K. oxytoca*				9			1	2	5	1				11.1
* K. pneumoniae*				61	17	17	17	8	1	1				83.6
* Providencia* spp.				2		2								100
* *rare fermenters				1		1								100
* Serratia* spp.				7			4	2	1					57.1
* *all				167	67	34	44	15	7	2				86.8
Unassigned, ceftazidime MIC 8-32 mg/L														
* Citrobacter* spp.				1				1						0.0
* E. coli*				10		1	1		1	1	2	4		20
* Enterobacter* spp.				5	1			1	1		2			20
* K. oxytoca*				3							1	2		0.0
* K. pneumoniae*				49			1	13	24	8	3			2.0
* *rare fermenters				1					1					0.0
* *all				69	1	1	2	15	27	9	8	6		5.8
Unassigned, ceftazidime MIC ≥64 mg/L														
* Citrobacter* spp.				2								1	1	0.0
* E. coli*				80					1	2	1	36	40	0.0
* Enterobacter* spp.				16						1		7	8	0.0
* K. oxytoca*				2								1	1	0.0
* K. pneumoniae*				204						9	7	86	102	0.0
* *all				304					1	12	8	131	152	0.0

BP, breakpoint; %S, percentage susceptible.

a Hard-matched, isolate’s antibiogram conforms precisely to expected phenotype for the mechanism; soft-matched, isolate’s antibiogram best matches the phenotype expected for the mechanism, but with minor discrepancies.

**Figure 1:**
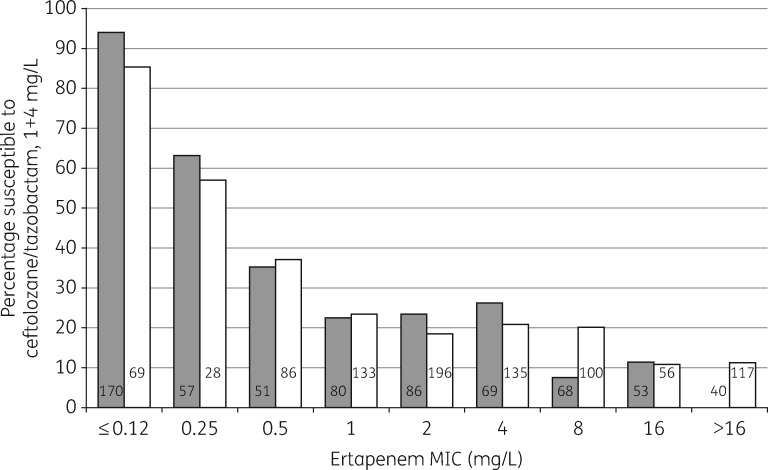
Susceptibility to ceftolozane/tazobactam among Enterobacteriaceae with ESBLs (grey bars, *n *=* *674) or raised AmpC (white bars, *n *=* *920) in relation to ertapenem MICs. Hard- and soft-matched isolates are included. None of the isolates included had a carbapenemase. Numbers on the bars indicate the numbers of isolates in the groups.

At its 1 + 4 mg/L breakpoint, ceftolozane/tazobactam was active against 41.7% of the ESBL producers, rising to 53.0% for *E. coli* and falling to 26.3% for *Klebsiella pneumoniae*. It was active also against 26.2% of AmpC hyperproducers, rising to 50.8% for *E. coli* and 88.9% for *Morganella morganii*, but falling to 18.4% for *Enterobacter* spp. The high susceptibility rate for AmpC-derepressed *M. morganii* reflects the vulnerability of *Morganella* AmpC to tazobactam,^7^ whilst frequent susceptibility in *E. coli* was associated with cefotaxime and ceftazidime MICs of 2–4 mg/L, a phenotype usually reflecting small elevations of chromosomal AmpC via promoter mutations.^8^ Frequent resistance among the AmpC *Enterobacter* spp. is in keeping with the fact that 554/649 (85.4%) of these referrals had high-level cefotaxime resistance (MIC ≥32 mg/L), as typically associated with total derepression of chromosomal enzyme. *Klebsiella* spp. have no chromosomal *ampC*, meaning AmpC phenotypes in this genus reflect acquired, plasmid-mediated enzymes: a 51.0% susceptibility rate accords with the observation that many examined by PCR (24/26) had *bla*_DHA_, a chromosomal ‘escape’ from *M. morganii* encoding a tazobactam-inhibited variant.^9^ Close relationships existed between ceftolozane/tazobactam MIC and ertapenem MICs for ESBL and AmpC producers, with the percentage of isolates susceptible to ceftolozane/tazobactam falling as ertapenem MIC increased (Figure 1). Previous experience indicates that raised ertapenem MICs for such carbapenemase-negative isolates mostly reflect porin loss.^10^^,^^11^

As with the BSAC series, ceftolozane/tazobactam was widely active (6/8 isolates susceptible) against *K. oxytoca* with high-level K1 enzyme, whereas activity against carbapenemase producers was very limited. All metallo-β-lactamase (MBL)-producing Enterobacteriaceae were resistant, as were almost all with KPC and GES-5 enzymes. On the other hand, 9/12 with rare class A carbapenemases (i.e. IMI, SME or FRI types) remained susceptible, probably because these enzymes are poor cephalosporinases. OXA-48-like carbapenemases are poor cephalosporinases too, especially against ceftazidime.^12^ On this basis, we categorized the OXA-48 producers as either ceftazidime-susceptible/intermediate (MIC ≤4 mg/L, EUCAST criteria) or ceftazidime-resistant (MIC >4 mg/L), inferring that the latter group had additional mechanisms such as ESBLs. Despite the presence of tazobactam (which should inhibit ESBLs), ceftolozane/tazobactam MICs closely tracked those of unprotected ceftazidime: thus, 81.9% of the ceftazidime-susceptible/intermediate isolates were susceptible to ceftolozane/tazobactam at 1 + 4 mg/L, versus 8.1% of ceftazidime-resistant isolates.

Ceftolozane/tazobactam was active against around 80% of Enterobacteriaceae isolates inferred to have reduced permeability without ESBL, AmpC or carbapenemase activity—a group also widely susceptible (MIC ≤1 mg/L, EUCAST) to cefotaxime (59.6%), ceftazidime (48.3%) and cefepime (56.2%). Ceftolozane/tazobactam also, unsurprisingly, had near-universal activity against cephalosporin-susceptible WTs. For isolates (mostly *K. pneumoniae*) with unassigned mechanisms, ceftolozane/tazobactam MICs tracked those of unprotected ceftazidime, with 86.8% susceptibility for isolates inhibited by ceftazidime at ≤4 mg/L, 5.8% for those with ceftazidime MICs of 8–32 mg/L and universal resistance among those with ceftazidime MICs ≥64 mg/L. It should, however, be stressed that these groups are diverse, and include several phenotypes under active investigation.

### P. aeruginosa

The two largest groups of referred *P. aeruginosa* were those inferred to have increased efflux or derepressed AmpC. The increased efflux group was subcategorized into those with carbenicillin MICs of 256–512 mg/L and those with carbenicillin MICs >512 mg/L (Table 4). MICs of ceftolozane/tazobactam increased in tandem with those of carbenicillin, piperacillin and ceftazidime for these isolates (Table 4); nevertheless, even among efflux-type isolates with carbenicillin MICs >512 mg/L, 94.7% remained susceptible to ceftolozane/tazobactam 4 + 4 mg/L, versus 27.6% for ceftazidime and 5.9% for piperacillin/tazobactam. Among efflux-type isolates with carbenicillin MICs 256–512 mg/L, all but one (99.7%) were susceptible to ceftolozane/tazobactam versus 65.3% for ceftazidime and 39.4% for piperacillin/tazobactam. Ceftolozane/tazobactam retained activity against 96.6% of *P. aeruginosa* inferred to have derepressed AmpC, whereas ceftazidime was active against only 20.8%, rising to 94.5% for ceftazidime/avibactam. Carbenicillin, which has good stability to pseudomonal AmpC,^13^ remained active against 84.6% of AmpC-derepressed isolates at its 128 mg/L breakpoint. Isolates with ‘WT’ carbenicillin MICs of 32–128 mg/L and without additional mechanisms affecting piperacillin/tazobactam or ceftazidime were split according to imipenem non-susceptibility (MIC >4 mg/L), which was taken as a marker of OprD loss; ceftolozane/tazobactam MICs were independent of this trait.

**Table 4:** MIC distributions of ceftolozane/tazobactam for all referred *P. aeruginosa* by assigned group and for other non-fermenters

	Match level				No. of isolates with indicated ceftolozane/tazobactam MIC, mg/L									%S at 4 + 4 mg/L
Category	molecular	hard^a^	soft^a^	total	≤0.25	0.5	1	2	4	8	16	>16	void	
*P. aeruginosa*														
AmpC hyperproducers		143	6	149	3	42	51	39	9	4	1			96.6
carbenicillin hypersusceptible (MIC ≤16 mg/L)		12	63	75	23	28	17	4	3					100.0
normal efflux, carbenicillin MIC 32–128 mg/L; imipenem MIC ≤4 mg/L		68	1	69	20	44	4		1					100.0
normal efflux, carbenicillin MIC 32–128 mg/L; imipenem MIC >4 mg/L		161	73	234	40	159	33	2						100.0
raised efflux, carbenicillin MIC 256–512 mg/L		262	136	398	7	182	158	41	9	1				99.7
high efflux, carbenicillin MIC >512 mg/L		126	26	152		21	66	37	20	7		1		94.7
ESBL producers	10^b^	21		31				1			2	28		3.2
GES carbapenemase producers	19			19					16	2		1		84.2
MBL producers	125			125							1	124		0.0
unassigned, ceftazidime MIC ≤8 mg/L				79	9	37	25	6	2					100.0
unassigned, ceftazidime MIC 16–128 mg/L				40		2	4	6	15	5	4	4		67.5
unassigned, ceftazidime MIC ≥256 mg/L				43					7	8	11	17		16.3
Other non-fermenters														
* A. baumannii*				335	12	9	2	13	37	42	68	152		no BP
* Acinetobacter* non-*baumannii*				84	44	10	7	6	5	1	1	10		no BP
* Achromobacter* spp.				61				2	3	3	11	42		no BP
* Burkholderia* spp.				76	14	14	16	8	9	5	2	8		no BP
* Chryseobacterium* spp.				9	2	1	2	3	1					no BP
* Elizabethkingia* spp.				26				1	5	6	10	4		no BP
* Pandoraea* spp.				24				1			1	22		no BP
* Pseudomonas* non-*aeruginosa*				86	24	23	14	5	3	2	7	7	1	no BP
* S. maltophilia*				78	9	16	19	10	3	5	2	14		no BP
others				31	7	1	2	2	1	3	2	13		no BP

BP, breakpoint; %S, percentage susceptible.

a Hard-matched, isolate’s antibiogram conforms precisely to expected phenotype for the mechanism; soft-matched, isolate’s antibiogram best matches the phenotype expected for the mechanism, but with minor discrepancies.

b *bla*_VEB_ confirmed by PCR; other ESBL producers were identified by phenotype.

In contrast to this good activity against isolates with elevated efflux, derepressed AmpC or loss of OprD, resistance to ceftolozane/tazobactam was near universal (96.8%–100%) among *P. aeruginosa* with MBLs or VEB ESBLs. Isolates with these enzymes also were broadly resistant to other penicillins and cephalosporins, with resistance rates >95%, though 72% of MBL producers remained intermediately susceptible to aztreonam according to EUCAST criteria, with MICs of 2–16 mg/L. Ceftolozane/tazobactam 4 + 4 mg/L was active against 16/19 GES carbapenemase-positive isolates, compared with 74% for ceftazidime 8 mg/L; however, 14 of these 19 were from a single outbreak, and the results may not be generalizable. Aside from MBL and ESBL producers, the only *P. aeruginosa* groups where ceftolozane/tazobactam resistance was frequent were the unassigned categories with ceftazidime MICs of 16–128 and ≥256 mg/L. Ceftolozane/tazobactam MICs for these isolates mostly were 8–16 mg/L, thus slightly lower than for MBL and ESBL producers.

Two wider associations were seen. First, across all 1414 *P. aeruginosa*, there was broad correlation between the MICs of ceftolozane/tazobactam and ceftazidime, with ceftolozane/tazobactam MICs 2–8-fold lower except for isolates with MBLs or ESBLs. Secondly, (Table 5) among 422 referred *P. aeruginosa* that were non-susceptible to all established β-lactams (i.e. carbenicillin MIC >128 mg/L, piperacillin/tazobactam >16 + 4 mg/L, ceftazidime >8 mg/L, imipenem >4 mg/L and meropenem >4 mg/L), 51.9% were susceptible to ceftolozane/tazobactam 4 + 4 mg/L, rising to 80.9% among the 267 lacking ESBLs, MBLs or GES enzymes.

**Table 5:** Activity of ceftolozane/tazobactam against *P. aeruginosa* non-susceptible to all other anti-pseudomonal β-lactams

	No. of isolates with indicated ceftolozane/tazobactam MIC (mg/L)									
	≤0.25	0.5	1	2	4	8	16	>16	Total	%S
All	0	20	90	72	37	23	15	165	422	51.9
Excluding carbapenemase and known ESBL producers	0	20	90	71	35	21	12	18	267	80.9

%S, percentage susceptible.

Non-susceptibility to other β-lactams defined as carbenicillin MIC >128 mg/L, ceftazidime MIC >8 mg/L, piperacillin/tazobactam MIC >16 mg/L and imipenem and meropenem MICs >4 mg/L, based on EUCAST breakpoints.

### Other non-fermenters

MIC distributions of ceftolozane/tazobactam for other non-fermenters besides *P. aeruginosa* are shown in Table 4. The *A. baumannii* submissions had a major bias towards carbapenem-resistant isolates, with 303/355 non-susceptible to imipenem at its 2 mg/L breakpoint, mostly (>95%) owing to OXA carbapenemases (not shown). Other species tend to be submitted owing to multiresistance, with many of the isolates from cystic fibrosis patients.

**Figure 2:**
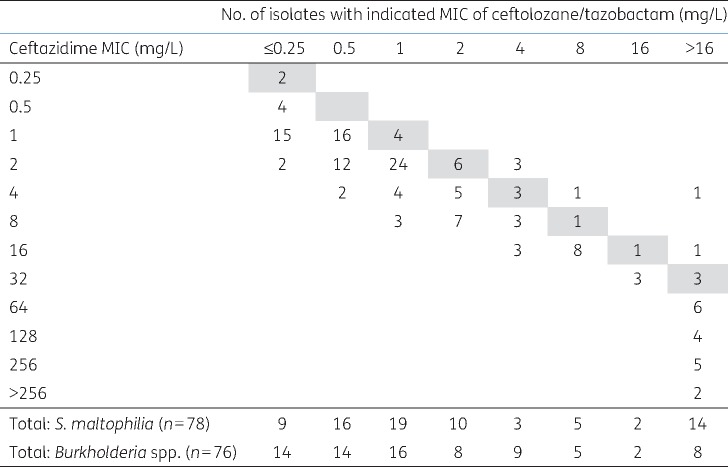
MICs of ceftolozane/tazobactam in relation to those of ceftazidime for *S. maltophilia* and *Burkholderia* spp., pooled. Grey squares indicate the line of equivalence, numbers below this line indicate that ceftolozane/tazobactam is more active than ceftazidime.

There are no ceftolozane/tazobactam breakpoints for non-fermenters besides *P. aeruginosa* but a few general points can be made. First, MICs for *A. baumannii*, *Elizabethkingia* spp. and *Pandoraea* spp. were mostly above the *P. aeruginosa* breakpoint of 4 + 4 mg/L, whereas MICs for non-*baumannii Acinetobacter*, *Burkholderia* spp., *Chryseobacterium* spp., non-*aeruginosa Pseudomonas* and *Stenotrophomonas maltophilia* were mostly at or below this breakpoint. Secondly, 29/32 imipenem-susceptible *A. baumannii* were susceptible to ceftolozane/tazobactam 4 + 4 mg/L versus only 44/303 imipenem-non-susceptible isolates. Thirdly, the MICs of ceftolozane/tazobactam for *S. maltophilia* and *Burkholderia* spp. correlated with those of ceftazidime, but were 2–4-fold lower (Figure 2).

## Discussion

Ceftolozane/tazobactam combines a new oxyimino-cephalosporin with an established β-lactamase inhibitor. It is licensed for complicated intra-abdominal and urinary tract infections, with efficacy including ESBL-producing Enterobacteriaceae up to the 1 + 4 mg/L breakpoint.^1^^,^^14^ Activity against *P. aeruginosa* is striking, but this species was poorly represented in the licensing trials. We reviewed the combination’s activity against: (i) consecutive bloodstream isolates collected in the BSAC Bacteraemia Surveillance; and (ii) ‘problem’ Gram-negative bacteria submitted to AMRHAI. The latter lack a denominator but provide a snapshot of organisms causing resistance concerns in the UK. For both collections we categorized isolates based on molecular data and interpretive reading.

The BSAC Bacteraemia Surveillance showed ceftolozane/tazobactam to be active against 91.5%–99.7% of the major Enterobacteriaceae species. Activity included 97.9% of ESBL *E. coli*, but only 84%–85% of ESBL-producing *Klebsiella* and *Enterobacter* spp. and 50% of AmpC-derepressed *Enterobacter*. The latter result is in keeping with tazobactam being a poor inhibitor of Enterobacter AmpC^7^ and ceftolozane being a substrate; high resistance rates were likewise seen for referred AmpC-derepressed *Enterobacter* (Table 3), and have been observed previously.^15^ The 15%–16% resistance rates for ESBL-producing *Enterobacter* and *Klebsiella* in the BSAC Bacteraemia Surveillance are more surprising, since ESBLs are inhibited by tazobactam. About half this resistance was borderline, with MICs of 2 mg/L, but MICs for the remainder ranged up to 256 mg/L, with resistance not obviously associated with ESBL type. Plausible explanations are that some ESBL-producing *Klebsiella* and *Enterobacter* had secondary β-lactamases or permeability lesions. Farrell *et al.*^16^ previously reported 12.1% ceftolozane/tazobactam resistance in ‘ESBL phenotype’ *E. coli* and 69.6% resistance in ‘ESBL phenotype’ *K. pneumoniae* from 41 medical centres in Europe, Turkey and Israel; these high resistance rates seem unrepresentative of the generality of UK and Irish ESBL producers and closer to results for referred problem isolates (Table 3). They may reflect (i) larger proportions of ESBL producers with secondary mechanisms in the countries surveyed, or (ii) inclusion of strains with KPC enzymes, which can give a false ‘ESBL phenotype’ in terms of cephalosporin/clavulanate synergy. Frequent resistance to ertapenem among referred ESBL and AmpC producers is probably due to impermeability, and, among these isolates, ceftolozane/tazobactam resistance was common (Figure 1).

Unlike the BSAC collections, the AMRHAI referrals provided numerous carbapenemase-producing Enterobacteriaceae, most of which proved resistant to ceftolozane/tazobactam. This is unsurprising for MBL and KPC producers as (i) MBLs are not inhibited by tazobactam and (ii) KPC enzymes hydrolyse penicillanic acid sulphones, mitigating inhibition.^17^ Better activity might be expected against isolates with OXA-48-like enzymes, which are poorly active against oxyimino-cephalosporins,^12^ with any cephalosporin resistance arising from secondary mechanisms, principally ESBLs, which should be inhibited by tazobactam. Yet, in reality, ceftolozane/tazobactam susceptibility was largely restricted to those OXA-48-like isolates that were susceptible or intermediate to unprotected ceftazidime (MIC ≤4 mg/L) and therefore are inferred to lack secondary β-lactamases. Similar behaviour (not shown) was seen for ceftazidime/clavulanate. Plausible explanations, deserving investigation, are: (i) many resistant isolates additionally had permeability lesions or multiple β-lactamases, thus overwhelming the tazobactam; or (ii) OXA-48-like enzymes inactivate tazobactam and clavulanate.

In contrast to this mixed performance against Enterobacteriaceae, ceftolozane/tazobactam was the most active β-lactam against both series of *P. aeruginosa* isolates. For the BSAC isolates its modal MIC was 4-fold lower than for ceftazidime and the proportion non-susceptible was only 0.2%. More strikingly, ceftolozane/tazobactam was active, at breakpoint, against 99.7% and 94.7% of referred isolates with moderately and strongly increased efflux and against 96.6% with derepressed AmpC. These are the most common resistance mechanisms in *P. aeruginosa* in the UK^18^^,^^19^ and Western Europe,^20–22^ though MBLs and ESBLs, which ceftolozane/tazobactam did not overcome, are more prevalent in Russia, Eastern Europe and the Middle East.^23–25^ Ceftolozane does not entirely escape efflux and AmpC, and its MICs are slightly raised, nevertheless it is less compromised than other cephalosporins and penicillins, and seems less prone to select for these traits.^26^

High-level ceftolozane/tazobactam resistance (MIC >16 mg/L) in *P. aeruginosa* was largely confined to isolates with MBLs or ESBLs, and these associations were so strong that AMRHAI now uses a ceftolozane/tazobactam MIC >16 mg/L as a predictor of these enzymes for the species. The lack of activity against ESBL-producing *P. aeruginosa* may seem surprising, since ESBL-producing Enterobacteriaceae were widely susceptible, but we note: (i) the predominant VEB ESBLs may be less susceptible to tazobactam than the CTX-M, TEM and SHV enzymes of Enterobacteriaceae; and (ii) tazobactam may be a poor permeant, or good efflux substrate, for *P. aeruginosa*. Piperacillin/tazobactam by analogy is poorly active against *P. aeruginosa* with acquired penicillinases.^3^

Ceftolozane/tazobactam offered no gain compared with earlier cephalosporins against *Acinetobacter* spp. or *Elizabethkingia* spp., but was more active than ceftazidime against *Burkholderia* spp. and *S. maltophilia*. β-Lactams remain controversial in infections due to these species, with medium-dependent MICs^27^ and no EUCAST breakpoints. Nevertheless they are often the next-most-active option after co-trimoxazole *in vitro*, and may be considered in sulphonamide-intolerant patients or those with co-trimoxazole-resistant strains. Ceftazidime, meropenem and temocillin have the lowest MICs among β-lactams for *Burkholderia* spp.,^28^ with ticarcillin/clavulanate and ceftazidime the most active against *S. maltophilia*.^29^ It now seems appropriate to add ceftolozane/tazobactam to this list. Activity against *S. maltophilia* may reflect tazobactam inhibiting the L-2 cephalosporinase, and this possibility deserves exploration.

In countries with conservative antimicrobial usage, such as the UK, new agents enter use as microbiologically directed treatments of problem infections, not as the empirical therapy modelled by Phase III trials. *P. aeruginosa* is the likely major target for ceftolozane/tazobactam, since β-lactams are core to anti-pseudomonal regimens and the combination inhibited many isolates resistant to all other β-lactams (Table 5). Results of a high-dosage ventilator-associated pneumonia trial, where *P. aeruginosa* is likely to be a frequent pathogen, are awaited with interest. In the meantime there is a growing catalogue of case reports of success in chronic and acute *P. aeruginosa* infections.^30–34^ These data are encouraging, although reports of resistance associated with AmpC sequence variants are a concern.^26^^,^^35^
